# Experimental design for efficient identification of gene regulatory networks using sparse Bayesian models

**DOI:** 10.1186/1752-0509-1-51

**Published:** 2007-11-16

**Authors:** Florian Steinke, Matthias Seeger, Koji Tsuda

**Affiliations:** 1 Max Planck Institute for Biological Cybernetics, Spemannstr. 38, 72076 Tübingen, Germany

## Abstract

**Background:**

Identifying large gene regulatory networks is an important task, while the acquisition of data through perturbation experiments (*e.g*., gene switches, RNAi, heterozygotes) is expensive. It is thus desirable to use an identification method that effectively incorporates available prior knowledge – such as sparse connectivity – and that allows to design experiments such that maximal information is gained from each one.

**Results:**

Our main contributions are twofold: a method for consistent inference of network structure is provided, incorporating prior knowledge about sparse connectivity. The algorithm is time efficient and robust to violations of model assumptions. Moreover, we show how to use it for optimal experimental design, reducing the number of required experiments substantially. We employ sparse linear models, and show how to perform full Bayesian inference for these. We not only estimate a single maximum likelihood network, but compute a posterior distribution over networks, using a novel variant of the expectation propagation method. The representation of uncertainty enables us to do effective experimental design in a standard statistical setting: experiments are selected such that the experiments are maximally informative.

**Conclusion:**

Few methods have addressed the design issue so far. Compared to the most well-known one, our method is more transparent, and is shown to perform qualitatively superior. In the former, hard and unrealistic constraints have to be placed on the network structure for mere computational tractability, while such are not required in our method. We demonstrate reconstruction and optimal experimental design capabilities on tasks generated from realistic non-linear network simulators.

The methods described in the paper are available as a Matlab package at

.

## Background

Retrieving a gene regulatory network from experimental measurements and biological prior knowledge is a central issue in computational biology. The DNA micro-array technique allows to measure expression levels of hundreds of genes in parallel, and many approaches to identify network structure from micro-array experiments have been proposed. Models include dynamical systems based on ordinary differential equations (ODEs) [1-5], Bayesian networks [6,7], or Boolean networks [8]. We focus on the ODE setting, where one or few expression levels are perturbed by external means, such as RNA interference [9], gene toggle switches (plasmids) [10], or using diploid heterozygotes, and the network structure is inferred from changes in the system response. So far only few studies investigate the possibility of designing experiments *actively*. In an active setting, *experimental design *is used to choose an order of perturbations (from a set of feasible candidates) such that maximum novel information about the underlying network is obtained in each experiment. Multi-gene perturbations are becoming increasingly popular, yielding more informative data, and automated data-driven design technologies are required to deal with the combinatorial number of choices which can be opaque even for a human expert.

Identifying (linear) ODE systems from observations and experimental design are well developed within the control community [11]. However, in the systems biology context, only very few measurements are available compared to the dimension of the system (*i.e*. number of genes), and experiments leading to such observations are severely restricted. Biological measurements are noisy, and time resolution is low, so that in practice only steady states of a system may be accurately measurable. On the other hand, there are no real-time requirements in biological control applications, and more advanced models and analysis can be used. A large body of biological knowledge can be used to counter the small number of observations, for example by specifying a prior distribution within a Bayesian method. The standard system identification and experimental design solutions of control theory may therefore not be well-suited for biology.

We propose a full Bayesian framework for network recovery and optimal experimental design. Given many observed genes and rather few noisy measurements, the recovery problem is highly under-determined, and a prior distribution encoding biological knowledge about the connectivity matrix does have a large impact. One of the key assumptions is network sparsity, which holds true for all known regulatory networks. We adopt the linear model frequently used in the ODE setting [1,2,4,5,12], but use a sparsity-enforcing prior on the network matrix. The sparse linear model is the basis of the *Lasso *[13], previously applied to the gene network problem in [12]. However, they simply estimate the single network maximizing the posterior probability from passively acquired data, and do not address experimental design. We closely approximate the Bayesian posterior distribution over connectivity matrices, allowing us to compute established design criteria such as the information gain, which cannot be done using maximum a posteriori (MAP) estimation. The posterior distribution cannot be computed in closed form, and obtaining an accurate approximation efficiently is challenging. We apply a novel variant of the recent expectation propagation algorithm towards this end.

Many other approaches for sparse network recovery have been proposed. In [1], the space of possible networks (as computed by a SVD) is scanned for the sparsest solution. A sparse Bayesian model is proposed in [14], see also [15]. While there is some work on experimental design for boolean networks [16] and Bayesian causal networks [17], none of the above mentioned methods have been used towards this goal. Experimental design remains fairly unexplored in the sparse ODE setting, with the notable exception of [3]. We compare our approach to theirs, finding our method to perform recovery with significantly less experiments and running much faster. Our method is more robust to observation noise frequently present for biological experiments, and somewhat more transparent and in line with statistical practice. Finally, their method consists of a combinatorial search and is therefore only applicable to networks with uniformly small in-degree, an assumption invalid for many known regulatory networks, *e.g*. [18].

## Results and Discussion

### Algorithm

#### Our Model

We start with the common linearized ODE model: expression levels ***x***(*t*) ∈ ℝ^*N *^of *N *measured genes at time *t *are modeled by the stochastic dynamical system

*d****x***(*t*) = ***f***(***x***(*t*))*dt *- ***u***(*t*)*dt *+ *d****W***(*t*).

Here, ***f***: ℝ^*N *^→ ℝ^*N *^describes the non-linear system dynamics, ***u***(*t*) is a user-applied disturbance, and *d****W ***(*t*) is white noise. With ***u***(*t*) ≡ **0**, we assume that the system settles in a steady state, and we linearize the system around that point. In this setting, a perturbation experiment consists of applying a constant ***u***(*t*) ≡ ***u***, then measuring the difference ***x ***between new and undisturbed steady state. Under the linearity assumption, we have that

***u ***= ***Ax ***+ *ε*,

where ***A ***is the *system matrix *with entries *a*_*ij*_, the non-zero *a*_*ij *_describing the gene regulatory network. The noise ***ε***is assumed to be i.i.d. Gaussian with variance *σ*^2^. We focus on steady state differences, as in [3]. Time course measurements are modelled linearly in [4,5], and our method can easily be formulated in their setup as well. We assume that the disturbances ***u ***do not drive the system out of the linearity region around the unperturbed steady state. While this seems a fairly strong assumption, our simulation experiments show that effective network recovery is possible even if it is partly violated.

Our contribution to this standard linear regression formulation is a Bayesian model, incorporating prior information about ***A***, namely its sparsity. The unknown matrix ***A ***is inferred via a posterior distribution, rather than merely estimated, allowing us to perform experimental design within a statistically optimal framework.

Observations are denoted ***X ***= (***x***_1 _... ***x***_*m*_)^*T*^, ***U ***= (***u***_1 _... ***u***_*m*_)^*T*^, and the Bayesian posterior is

*P*(***A***|***U***, ***X***) ∝ *P*(***U***|***A***, ***X***)*P*(***A***),

where the likelihood is $P(U|A,X)=\prod_{j=1}^{m}N(u_{j}|Ax_{j},\sigma^{2}I)$, owing to (2).

Note that typically *m < N*, certainly in early stages of experimental design, and ***U ***= ***XA ***has no unique solution. In this situation, the encoding of knowledge in the prior *P*(***A***) is of large importance. True biological networks are known to be sparsely connected, so we would expect sparse network matrices ***A***. The prior should force as many entries of ***A ***close to zero as possible, at the expense of allowing for fairly large values of a few components. It should be a *sparsity prior*.

We employ a *Laplace *prior distribution

$$\begin{array}{cc} P(A)=\prod_{i,j}P(a_{ij}), & P(a_{ij})=\frac{\tau }{2}{\text{e}}^{-\tau \left|a_{ij}\right|}. \end{array}$$

**Figure 1:**
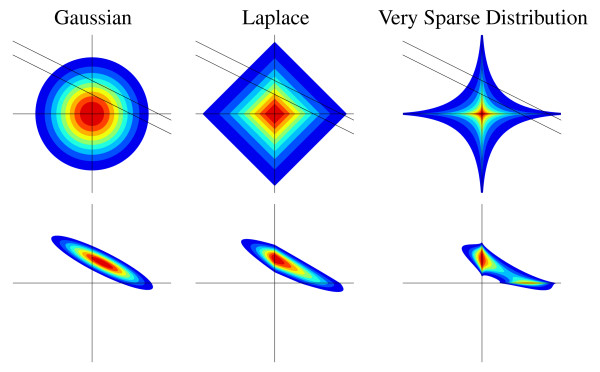
**The Choice of Model**. Three prior distribution candidates over network matrix coefficients: Gaussian, Laplace, and "very sparse" distribution (*P*(*a*_*ij*_) ∝ exp(- *τ*|*a*_*ij*_|^0.4^)). We show contour plots of density functions over two entries, coloured areas contain the same probability mass for each of the distributions. Upper row: prior distributions (unit variance), and likelihood for single measurement (linear constraint with Gaussian uncertainty). Lower row: corresponding posterior distributions. The Gaussian is spherically distributed, the others shift probability mass towards the axes, giving more mass to sparse tuples (≥ 1 entry close to 0). This effect is clearly visible in the posterior distributions. For the Gaussian prior, the area close to the axes has rather low mass. The Laplace-posterior is skewed: more mass is concentrated close to the vertical axis. Both posteriors are log-concave (and unimodal). The "very sparse"-posterior is shrunk towards the axes more strongly, sparsity is enforced stronger than for the Laplace prior. But it is bimodal, giving two different interpretations for the single observation. This multimodality increases exponentially with the number of dimensions, rendering accurate inference very difficult. The Laplace prior therefore is a good compromise between computational tractability and suitability of the model.

It is instructive to compare the Laplace against the Gaussian distribution, which is commonly used as prior in the linear model. The Laplace puts much more weight close to zero than the Gaussian, while still having higher probabilities for large values. The implications are depicted in Figure 1, see also [15]. In fact, the Gaussian prior is used with the linear model mostly for convenience, since the posterior is Gaussian again and can be computed easily [19]. Even within our framework, computations with a Gaussian prior are significantly more efficient than with a Laplace. However, our results prove that theoretical arguments in favour of the Laplace prior do have real practical weight, in that the computational advantages with the Gaussian are paid for by a much worse predictive accuracy, and identification needs significantly more measurements than for the Laplace.

The bi-separation characteristic of the Laplace prior into few large and many small parameters (which is not present for the Gaussian) is embodied even more strongly in other sparsity priors, such as "spike-and-slab" (mixture of narrow and wide Gaussian), Student-*t*, or distributions based on *α*-norms, ${\left\|x\right\|}_{\alpha }^{\alpha }=\sum_{i}{\left|x_{i}\right|}^{\alpha }$, with *α *< 1, see also Figure 1. However, among these only the Laplace distribution is log-concave, i.e. has a log-concave density function, leading to a posterior whose log density is a concave function, thus has a single local maximum. This simplifies accurate inference computations significantly. For a non-log-concave prior, posteriors are usually multi-modal, spreading their mass among many isolated bumps, and the inference problem is in general at least as hard as the combinatorial problem of testing all possible sparse graphs. For such posteriors, all known methods for approximate Bayesian inference tend to either perform poorly or require an excessive amount of time. Furthermore, they tend to be algorithmically unstable, and the approximation quality is hard to assess. Robustness of the inference approximation is important for experimental design, since decisions should not be based on numerical instability artefacts of the method, but on the data alone. These points motivate our choice of a Laplace sparsity prior.

Note that the Laplace prior does not imply any strict constraints on the graph structure, *i.e*. the sparsity pattern of ***A***, in contrast to other combinatorial approaches which can be run affordably only after placing hard constraints on the in-degree of all network nodes [3]. The Laplace prior *P*(***A***) and the resulting posterior have densities, so that the probability of a matrix ***A ***having entries exactly equal to zero vanishes. Sparsity priors with point masses on zero have been used in Statistics, but approximate Bayesian inference for such is very hard in general (such priors are certainly not log-concave). We predict discrete network graphs from our posterior as follows. For a small threshold *δ*_*e*_, we take *a*_*ij *_to represent an edge *i *← *j *iff |*a*_*ij*_| > *δ*_*e*_. Moreover, the marginal posterior probability of {|*a*_*ij*_| > *δ *_*e*_} is used to rank potential edges *i *← *j*.

The posterior for the sparse linear model with Laplace prior does not fall into any standard multivariate distribution family, and it is not known how to do computations with it analytically. On the other hand, experimental design requires a good approximation to the posterior, which can be updated efficiently in order to score an experiment. Denote the observations (experiments) obtained so far by *D*. From (3) and (4), we see that the posterior factorizes w.r.t. rows of ***A***, in that $P(A|D)=\prod_{i}P(A_{i,}^{T}.|D)$, where $A_{i,}^{T}.$ is the *i*-th row of ***A***. The factors are joint distributions over *N *variables. We noted above that these factors are log-concave, and thus have a single local maximum and convex upper level sets (see Figure 1). These features motivate approximating them by Gaussian factors, so that a posterior approximation is obtained as *Q*(***A***) = ∏_*i *_*Q*($A_{i,}^{T}.$) with multivariate Gaussians *Q*($A_{i,}^{T}.$). The approximate inference method we use is a novel variant of *expectation propagation *(EP) [20,21]. Our approach deals correctly with very underdetermined models (*m *≪ *N *in our setup), where previous EP variants would fail due to severe numerical instability. Details are provided in the Methods section, see also [22].

#### Experimental Design

In our setup, an experiment consists of applying a constant disturbance ***u ***to the system, then measuring the new steady state. With current technology, such an experiment is expensive and time-consuming, especially if ***u ***is to be controlled fairly accurately. The goal of sequential experimental design is to choose the next experiment among a set of candidates (of about the same cost), with the aim of decreasing the uncertainty in ***A ***using as *few experiments as possible*. A successful design methodology allows to obtain the same conclusion with less cost and time, compared to doing experiments at random or even following an exhaustive coverage. To this end, an information value score is computed for each candidate, and the maximizer is chosen.

Different costs of experiments can be considered by multiplying the information value score with the costs. However, note that if the costs are extremely different, experiment design is often not necessary since the costs alone determine what should be done next.

A straightforward choice of an information value score is the expected decrease in uncertainty. In general, experimental design thus cannot be done without a representation of uncertainty in ***A***, and the Bayesian framework maintains such a representation at its core, namely the posterior. Methods based solely on maximum likelihood or maximum a posteriori estimation (such as Lasso) fail to represent uncertainties. Denote the current posterior by *Q*(***A***) = *Q*(***A***|*D*). If (u_*_, x_*_) is the outcome of an experiment, let *Q'*(***A***) = *Q'*(***A***|*D *∪ {(u_*_, x_*_)}) be the posterior including the additional observation. Different information value scores have been proposed for experimental design, see [23] for an overview. A measure for the amount of uncertainty in *Q *is the differential entropy E_*Q *_[- log *Q*], so a convenient score would be the entropy difference E_*Q *_[- log *Q*] - E_*Q' *_[- log *Q'*]. A related score is the *information gain S*(u_*_, x_*_|*D*) = D[*Q' *|| *Q*] = E_*Q' *_[log *Q' *- log *Q*]. Here, D[*Q' *|| *Q*] is the relative entropy (or Kullback-Leibler divergence), a common measure for the "cost" (in terms of information) of replacing *Q' *by *Q*. The inclusion of a new experiment leads precisely to the replacement *Q *→ *Q'*, so the information gain is well-motivated in our setup. While scores such as information gain or entropy difference are hard to compute for general distributions *Q*, *Q'*, this can be done straightforwardly for Gaussians. If *Q*(***a***) = *N*(***h***, **Σ**), *Q'*(***a***) = *N*(***h'***, **Σ'**) and ***a ***= $A_{i,}^{T}.$, the information gain is

$$\frac{1}{2}\left(\log\left|M\right|+\text{tr }M^{-1}-N+{\left(h^{\prime }-h\right)}^{T}\Sigma^{-1}(h^{\prime }-h)\right),$$

with ***M ***= (**Σ'**)^-1^**Σ**, which can be computed very efficiently in our framework.

The outcome (u_*_, x_*_) of an experiment is of course not completely known before it is performed. The central idea of Bayesian sequential design is to compute the distribution over outcomes of the experiment, based on all observations so far, with which to average the score *S*(u_*_, x_*_|*D*). Thus, some experimental candidate *e *is represented by a distribution *Q*_*e*_(· |*D*) over (u_*_, x_*_). In the setting of this paper, u_* _is completely known, say u_* _= ***u***^(*e*) ^for candidate *e*, although in an extended setting, *e *might only specify a distribution over u_*_. Given u_* _= ***u***^(*e*)^, $Q_{e}(u_{*},x_{*}|D)={\text{I}}_{\left\{u_{*}=u^{\left(e\right)}\right\}}Q(x_{*}|D,u_{*})$, which can be sampled from easily: first, draw ***A ***~ *Q*(***A***|*D*), then x_* _= ***A***^-1^(u_* _- ε_*_), ε_* _~ *N*(**0**, *σ*^2^***I***). In general, the information value for candidate *e *is given as $S(e|D)={\text{E}}_{Q_{e}}[S(u_{*},x_{*}|D)]$, which specializes to $S(u^{\left(e\right)}|D)=S(u_{*}|D)={\text{E}}_{Q(x_{*}|D,u_{*})}[D[Q^{\prime }||Q]]$ in our setup here.

### Testing

In the literature, there are some small networks with known dynamics, *e.g*. the Drosophila segment polarity network [24]. However, a thorough evaluation of our method requires significantly larger systems for which the dynamics are known, so that disturbance experiments can be simulated, and the predictions of our method can be verified. We are not aware of such models having been established for real biological networks yet, the DREAM project [25] aims at providing such data in the future. We therefore concentrate on realistic "in-silico" models, applying our method to many randomly generated instances with different structures and dynamics in order to obtain a robust evaluation and comparison.

We simulate the whole network identification process. First, we generate a biologically inspired ground-truth network together with parameters for a numerical simulator of nonlinear dynamics. We feed our method with a number of candidate perturbations {u_*_}, among which it can choose the experiments to be done. If some u_* _is selected, the corresponding x_* _is obtained from the simulator, and (u_*_, x_*_) is included into the posterior as new observation. We score the current posterior *Q*(***A***) against the true network after each inclusion, comparing our method against variants in different settings. Free hyperparameters (*τ*, *σ*^2^) are selected individually for each of the methods to be compared (see Methods section). We also compare against the experimental design method proposed in [3], and finally show results on the real, but small Drosophila segment polarity network [24].

#### Network Simulation

**Figure 2:**
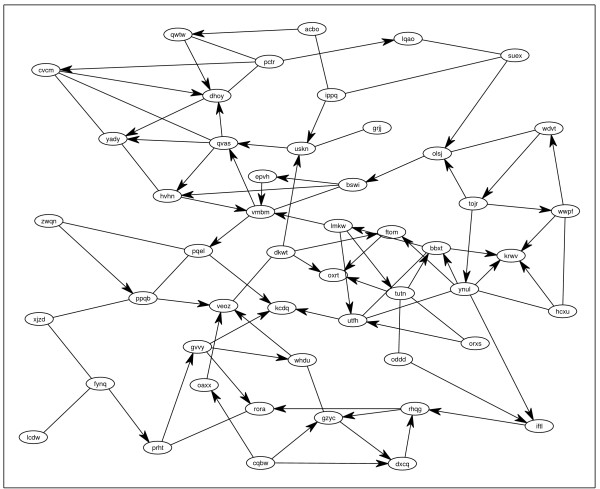
**An Example Network**. Small-world network of *N *= 50 nodes. Arrowless edges are bi-directional. "Gene names" are randomly drawn. Some nodes have rather high in-degree, characteristic of real biological networks, *e.g*. [18].

Common computational models of sparse regulatory networks often build on the *scale-free *or the *small-world *assumption [26]. In small world networks the average path length is much shorter than in a uniform random network. We sample such small-world networks with *N *= 50 nodes (unless otherwise said), see Figure 2 for an example. Further details about network generation and properties are given in additional file 1.

For a given network structure, we sample plausible interaction dynamics using Hill-type kinetics, inspired by the model in [2]. The non-linear function in (1) is

$$\begin{array}{lll} f_{i}(x) & = & -V_{di}\frac{x_{i}}{d_{i}+x_{i}}\\ & & +V_{si}\prod_{j\in A_{i}}\frac{1+A_{ij}{\left(\frac{x_{j}}{\kappa_{ij}}\right)}^{n_{ij}}}{1+{\left(\frac{x_{j}}{\kappa_{ij}}\right)}^{n_{ij}}}\prod_{j\in {ℐ}_{i}}\frac{1}{1+{\left(\frac{x_{j}}{\kappa_{ij}}\right)}^{n_{ij}}}, \end{array}$$

where $A_{i}({ℐ}_{i})$ are the activating (inhibitory) parents of gene *i*. The parameters in (6) and the way they are randomly sampled are described in additional file 1. Proposed system equations are subject to the condition, that the model produces dynamics with a reasonable stable steady state.

Each observation (***u***, ***x***) consists of a constant disturbance ***u ***and its effect ***x***, being the difference between a new (perturbed) and the old (unperturbed) steady state. Disturbance candidates were restricted to a small number *r *of non-zero entries, since experimental techniques for disturbing many genes in parallel by tightly controlled amounts are not yet available. All non-zero *u*_*j *_are in {±*ν*}, where the sign is random, so ||***u***|| is the same for all ***u***. We measure ||***u***|| in units given by the average relative change in steady state when such disturbances ***u ***are applied. We use a pool of 200 randomly generated candidates. The SDE simulator can be used with different levels of noise, measured in terms of the signal-to-noise ratio (SNR), *i.e*. the ratio of ||***u***|| and the standard deviation of the resulting *ε *in (2).

All results are averaged over 100 runs with independently drawn networks. In the comparative plots presented below, the different methods all see the same data in each run.

#### Evaluation Criterion

The output from a regulatory network identification method most relevant to a practitioner is a ranking of all possible links, ordered by the probability that they are true edges. With this in mind, we choose the following evaluation score, based on ROC analysis.

At any time, our method provides a posterior *Q*(***A***), of which at present we only use the marginal distributions *Q*(*a*_*ij*_). We produce a ranking of the edges according to the posterior probabilities *Q*({|*a*_*ij*_| > *δ*_*e*_}), where *δ*_*e *_= 0.1 in all experiments. *δ*_*e *_was calibrated against average component sizes |*a*_*ij*_|, which are roughly given through the dominant time scales in the dynamical system. The predicted rankings are robust against moderate changes of *δ*_*e*_.

In a standard ROC analysis, the true positive rate (TPR) is plotted as a function of the false positive rate (FPR), and the area under this curve (AUC) is measured. This is not useful in our setting, because only very small FPRs are acceptable at all (there are *N*^2 ^potential edges). Our *iAUC *score is obtained by computing AUC only up to a number of FP equal to the number of edges in the true network, normalized to lie in [0, 1]. For *N *= 50, the "baseline" of outputting a random edge ranking has an expected iAUC of 0.02. Furthermore, on average about 25% of the true edges are "undetectable" by any method using the linearized ODE assumption: although present in the nonlinear system, their entries *a*_*ij *_are very close to zero, and they do not contribute to the dynamics within the linearization region. Such edges were excluded from the computation of iAUC, for all competing methods.

## Discussion

**Figure 3:**
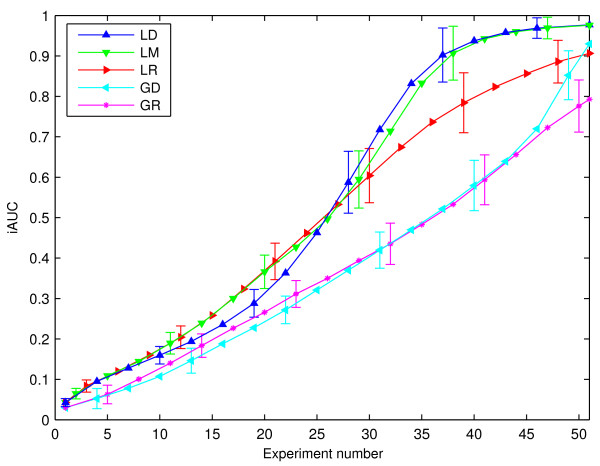
**Reconstruction Performance for Different Methods**. Reconstruction curves for experiments (gene expression changes of 1%, SNR 100, *r *= 3 non-zeros per ***u***). **LD**: Laplace prior, experimental design. **LR**: Laplace prior, random experiments. **GD**: Gaussian prior, experimental design. **GR**: Gaussian prior, random experiments. **LM**: Laplace prior, mixed selections (first 20 random, then designed). Error bars show one standard deviation over runs. All visually discernible differences in mean curves of different methods are significant under the *t*-test at level 1%.

In Figure 3, we present reconstruction curves for our method versus competing techniques, lacking novelties of our approach (optimal experimental design, Laplace sparsity prior). Very clearly, optimal design helps to save on costly and time-consuming experiments. The effect is more pronounced for the Laplace than for the Gaussian prior. The former is a better prior for the task, and it is well known that the advantage of designed versus random experiments scales with the appropriateness of the model. In this case, the iAUC level 0.9 is attained after 36 experiments with designed disturbances, yet only after 50 measurements with randomly chosen ones, thus saving 30% of the experiments.

In general, the model with Laplace prior does significantly better than with a Gaussian one (*τ *of the Laplace and the variance of the Gaussian prior were of course selected independently). The difference is most pronounced at times when significantly less than *N *experiments have been done and the linear system (2) is strongly under-determined. This confirms our arguments in favour of the Laplace prior.

The systematic underperformance of the most direct variant LD of our method, up to about *N/*2 observations, is not yet completely understood. One should be aware that aggressive experimental design based on very little knowledge can perform worse than a random choice. This is a variant of the well-known "explore-exploit" trade-off [27], which can be countered by either specifying prior knowledge more explicitly, or by doing a set of random inclusions (explore) before starting the active design (exploit). This is done in the LM variant.

**Figure 4:**
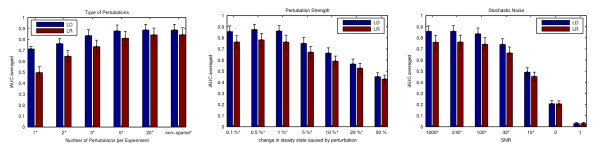
**Reconstruction Performance for Different Experimental Conditions**. Comparison between **LD **(Laplace, design) and **LR **(Laplace, random experiments) under different conditions. Score is average iAUC after 25, ..., 50 experiments. (Left): Number *r *of non-zero ***u ***coefficients in each disturbance varied, keeping ||***u***|| constant. (Middle): Norm ||***u***|| of disturbances varied, while keeping *r *= 3 and low noise level. (Right): Stochastic noise in the data (1) varied, for constant ||***u***||, *r *= 3. Settings marked with *: LD is significantly superior to LR, according to *t*-test at level 1%.

In Figure 4, experimental design is compared to the random experiment choice setting, both with a Laplace prior. In the left panel, we vary the number *r *of non-zero entries in the disturbances ***u***. Recall that large *r *are in fact unrealistic in experimental techniques available today, but may well become accessible in the future. The less constraints there are on ***u***, the more information one may obtain about ***A ***in each experiment, and the better our method performs. This is in line with linear systems theory, where *persistent excitations *[11] (*i.e*. full ***u***'s) are known to be most effective for exploring a system. The edge of experimental design is diminished with larger *r*. This is plausible, in that the informativeness of each ***u ***increases strongly with more non-zeros, thus the relative differences between ***u***'s are smaller. Experimental design can outperform random choices only if there are clear advantages in doing certain experiments over others.

The middle panel in Figure 4 explores effects of different sizes ||***u***||, *i.e*. different perturbation strengths (here, *r *= 3, and the noise in the SDE is very small). For larger ||***u***||, the real non-linear dynamics deviate more and more from the linearized ones, thus decreasing recovery performance above about 5%. On the other hand, larger ||***u***|| would result in a better SNR for each experiment, given that non-linear effects could be modelled as well. This is not yet done in our method, but these shortcomings are shared by all other methods relying on a linearization assumption. It is, however, encouraging that our method is quite robust to the fact that even at smaller ||***u***||, the residuals *ε *behave distinctly non-Gaussian (occasional large values).

The right panel in Figure 4 shows how increasing stochastic noise in (1) influences network recovery. We keep *r *= 3 and set ||***u***|| to generate steady state deviations of 1%. Good performance is obtained at SNRs beyond 10. With a SNR of 1, one cannot expect any decent recovery with less than *N *measurements. At all SNRs shown, the network was recovered eventually with more and more experiments, but this is probably not an option one has in current biological practice.

### Comparison to Tegnér *et.al*

The method proposed in [3] is state-of-the-art for experimental design applied to gene network recovery, and in this section, we compare our method against theirs. Their approach can be interpreted in Bayesian terms as well, this is detailed in additional file 1.

In contrast to our method, they discretize the space of possible matrices ***A***. Observations are used to sieve out candidates which are not "consistent" with all measurements so far. They have to restrict the maximum node in-degree for each gene to 3 in order to arrive at a procedure of reasonable cost. To our knowledge, the code used in [3] has not been released. We implemented it, following all details in their paper carefully (some details of our re-implementation are given in additional file 1). In general, the diagonal of ***A ***(self-decay rates) is assumed to be known in [3]. For the comparison, we modified our method to accept a fixed known diag ***A ***and changed the iAUC score not to depend on self-edges.

**Figure 5:**
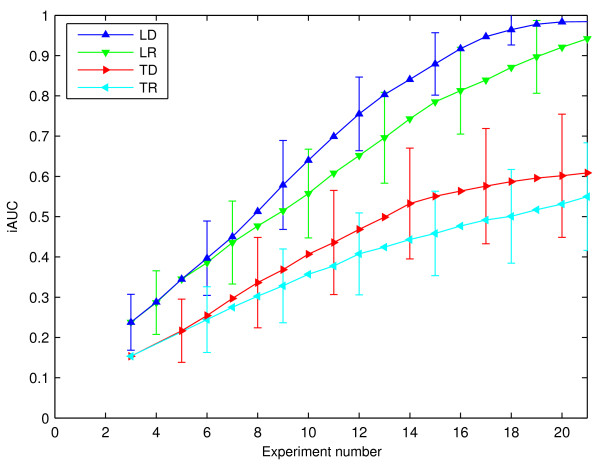
**Reconstruction Performance Compared to Tegnér *et. al***. Network recovery performance, comparing our method (Laplace, design) with [3]. Networks of size *N *= 20, *r *= 1 non-zeros in ***u***, perturbation size 1%, SNR 100. Three initial random experiments, to reduce memory requirements in [3] method. **TD**: [3], experimental design. **TR**: [3], random experiments. **LD**: Our method, Laplace prior, experimental design. **LR**: Our method, Laplace prior, random experiments.

Results of a direct comparison are shown in Figure 5, with and without the proposed optimal design methods. Due to the high resource requirements of the method in [3], we use networks of size *N *= 20 (simulated as above), restricted to in-degrees at most 3. In general, our method performs much better in recovering the true network. This difference is robust even to significant changes in the ground truth simulator. We find that their method is very sensitive to measurement and system noise, or to violations of the linearization assumption, whereas our technique is markedly more robust w.r.t. all these. We give some arguments why this might be the case. Firstly, their "consistency" sieve of ***A ***candidates in light of measurements is impractical. After every experiment a number of inconsistent ***A ***is rejected from consideration, and noisy experiments may well lead to a wrong decision. Any future evidence for such a rejected solution is, however, not considered any more. At the same time, an experiment does not help to discriminate between matrices which are still consistent afterwards. Another severe problem with their approach lies in the discretization of ***A ***entries. A histogram of values of *a*_*ij *_from our simulator reveals a very non-uniform (and also non-Gaussian) distribution: many values close to zero, but also a substantial number of quite large values. At the very least, their quantization would have to be chosen non-uniformly and adaptively, such that each bin has about equal mass under this distribution. However, it is quite likely that the best quantization depends on details of the true system which are not known *a priori*. Statistics with continuous variables, as we employ, is a classical way of avoiding such quantization issues. Furthermore, our Laplace prior seems to capture features of the *a*_*ij *_distribution favourably.

In Table 1, we compare running times. Even though they restrict the node in-degree to 3, which is often unrealistic for known biological networks [18], the required running times are orders of magnitude larger than for our method. Also, their memory requirements are huge, so that networks sizes beyond *N *= 50 could not be dealt with on a unit with 4 GB RAM. Both are clearly consequences of their quantization approach, which we circumvent completely by applying a continuous model. The asymptotic running time for a naive implementation of our method is *O*(*N*^5^) (Laplace, experimental design, *N *experiments), independent of the true network structure, but this can be reduced to *O*(*N*^4^) as discussed in the Methods section.

**Table 1:** Runtimes. Running time for full network recovery, comparing our method (Laplace, design) with [3]

N	20	30	40	50	100	150	200
Our method	0.02	0.08	0.2	0.5	8	52	175
Tegnér *et.al*. [3]*	0.8	5	16	55	-	-	-

In minutes; 2 GHz Opteron processor, 1.5 GB RAM. *: We allowed 4 GB RAM for [3], but this failed due to even higher demand for *N *> 50.

#### Drosophila segment polarity network

In [24], von Dassow *et.al*. describe a realistic model of the Drosophila segment polarity network. We tested our algorithm on a single cell submodule, using the equations and parameters as described in [3, Supplement], who also used this model.

So far, we modelled only mRNA levels. However, the Drosophila network also contains 5 proteins which play an important role in the regulatory network. Since proteins are hard to control and to observe, we treat them as unobserved variables and focus on identifying the *effective *network between the genes. A link *i *→ *j *between genes *i *≠ *j *in the effective network represents one or more interactions of the form *i *→ *P*_1 _→ ⋯ → *P*_*q *_→ *j*, where *P*_1_, ..., *P*_*q*_, *q *≥ 0 are intermediate proteins, but not genes. In the methods section, we give a mathematical proof that any method working on the observed part of the system only, such as ours, in fact focusses on identifying the effective network, given that the linearized ODE assumption is applied to the complete system. This is reassuring, since all regulatory networks between genes are nothing else but effective networks of larger partially unobserved systems.

**Figure 6:**
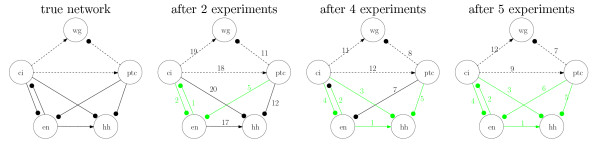
**Reconstruction of Drosophila segment polarity network**. The left figure shows the effective single cell model with five genes of the Drosophila segment polarity network [24]. Lines with circles denote inhibitory, arrows activating influence, functionally weak links are dashed. The figures on the right show the ranks that our algorithm assigns to each of the edges after *n *experiments (*n *= 2, 4, 5). There are 6 rel. strong edges with $\tilde{A}$_*ij *_≠ 0 in the network, and we assume that an edge is correctly identified if its rank is amoung the top 6. These edges are coloured green.

As shown in Figure 6, the network contains 9 inter-gene regulatory pathways, apart from the self-links that are dominated by the respective self-decay rates. Three of the inter-gene links are functionally weak (i.e. $\tilde{A}$_*ij *_≈ 0). We simulated single gene perturbation experiments with an ordering chosen by our algorithm (Laplace prior distribution, perturbation size 1%, SNR 100). After each experiment we ranked potential edges according to their probability. Resulting ranks after 2, 3, 5 experiments for the true network edges are shown in Figure 6. All significant network edges are recovered after 5 experiments (*iAUC *= 1). Even weak links are assigned low ranks compared to a maximal rank 20, which places them amoung the first that would have to be examined more closely.

## Conclusion

We have presented a Bayesian method for identifying gene regulatory networks from micro-array measurements in perturbation experiments (*e.g*., RNAi, toggle-switch, heterozygotes), and shown how to use optimal design in order to reconstruct networks with a minimum number of such experiments. The approach proves robust and efficient in a realistic non-linear simulation setting. Our main improvements over previous work consist of employing a Laplace prior instead of a simpler Gaussian one, encoding the key property of sparse connectivity of regulatory networks within the model, and of actively designing rather than randomly choosing experiments. Both features are shown to lead to significant improvements. When it comes to experimental design, our method outperforms the most prominent instance of previous work significantly, both in higher recovery performance and in smaller resource requirements. Our application of the recent expectation propagation technique to the under-determined sparse linear model is novel, and variants may be useful for other models in Bioinformatics.

In this paper, we have focussed on modelling mRNA levels, which can be measured easily and cost-effectively. However, protein and metabolite concentrations also play important roles in any regulatory pathway, and a concise ODE explanation of a system can probably not be formulated if they are ignored. Our method allows to treat these as unobserved variables and to identifying effective networks between the genes. However, if the additional variables can be directly measured, they can easily be treated explicitly within our method, by simply extending the state variable ***x***(*t*).

Throughout the paper we have assumed that u_* _is known for an experiment, *i.e*. the disturbance levels of the *r *targeted genes can be controlled or at least predicted in advance, before the experiment is actually done. For example, a study trying to model the efficacy of RNAi experiments is given in [28]. In the context of experiment design, we can only hope to compute the expected decrease in uncertainty for a specific experiment, and thus rank potential experiments according to their expected value, if the experimental outcome is predictable to some degree. In our method, the outcome x_* _for a given u_* _is inferred through the current posterior, *i.e*. the information gain from (u_*_, x_*_) is averaged over *Q*(x_*_|u_*_, *D*). This can be extended to uncertain u_*_, if distributions *Q*_*e*_(u_*_|*D*) specific to each experiment *e *can be specified. For experimental biology, this means that not only do we need experimental techniques which deliver quantitative measurements, but furthermore the parameters distinguishing between different experiments (***u ***in our case) either have to be fairly tightly controlled (our assumption in this paper), or their range of outcome has to be characterized well by a mathematical model.

In general, biological prior knowledge about the (effective) regulatory network may already be available before any experiments are done. In fact, in the presence of many genes *N*, it is typically not affordable to do on the order of *N *disturbance experiments, which are required for complete network identification in the absence of specific prior knowledge (it has been conjectured that *O*(log *N*) experiments are required only in [3], but we cannot confirm such a surprisingly fast scaling based on our experiments, even when using their method). Within our method, such prior knowledge can be incorporated if it can be formulated in terms of the system matrix ***A***. No interaction *i *← *j *is encoded as *a*_*ij *_= 0, an activating influence *i *← *j *as *a*_*ij *_> 0. These types of knowledge can be included in our method, as is discussed in the Methods section.

There are several other setups of formulating the network recovery problem in terms of a sparse linear model. Time-course mRNA measurements with unknown, yet time-constant disturbances ***u ***are used in [5] and [4]. Relative rather than absolute changes in expression levels are employed in [2]. Within all these setups, our general efficient Bayesian framework for the sparse linear model could be beneficial, and could lead to improvements due to the Laplace sparsity prior.

The linearized ODE assumption is frequently done [1-5,12], yet it is certainly problematic. For disturbances which change steady state expression levels by more than about 5%, our simulator showed a behavior which cannot directly be captured by a linearized approach. But such perturbation levels may be necessary to achieve a useful SNR in the presence of typically high measurement noise. An important point for future work is the extension of the model by simple non-linear effects of relevance to biological systems. For example, our model can directly be extended to higher-order Taylor expansions of non-linear dynamics, since these are still linear in the parameters.

## Methods

### Approximate Bayesian Inference

In this section, we provide an exposition and some possible extensions of our approximate inference method. We sketch the expectation propagation (EP) method for inference in the sparse linear model. Further details are given in [29].

Our aim is to approximate *Bayesian inference*, given a model and prior distributions for all unknowns. The *likelihood function *is the probability of the observed data, given all unknowns, it is determined entirely by the model. In our case, we have a Gaussian likelihood $P(D|A)=\prod_{j=1}^{m}N(u_{j}|Ax_{j},\sigma^{2}I)$, since the observation noise is assumed to be Gaussian. In the Bayesian framework, knowledge and assumptions about unknowns can be formulated in the model or in the prior distributions. In general, the model is used to specify knowledge which is given in an explicit deterministic form. In our example, the linearlzation assumption leads to the linear model. Prior distributions are used in order to formulate implicitly defined or non-deterministic properties. For example, we argued that since biological regulatory networks are sparsely connected, the matrix ***A ***should be sparse. If we knew *a priori *which entries of ***A ***are zero, we would modify the model by eliminating these components. The knowledge that many components should be close to zero, but the precise set of relevant components is unknown up front, is of a non-deterministic kind and is conveniently represented by the Laplace prior *P*(***A***).

The *posterior distribution *is

$$P(A|D)=P{\left(D\right)}^{-1}P(D|A)P(A)=\prod_{i=1}^{N}P(A_{i,}^{T}.|D)$$

by the rules of conditional probabilities. It factorizes w.r.t. rows of ***A***, since both prior and likelihood do. For the sparse linear model, computations based on the posterior factor *P*($A_{i,}^{T}.$|*D*) cannot be done analytically. In EP, the factor is approximated by a joint Gaussian *Q*($A_{i,}^{T}.$), with the aim of fitting mean and covariance of the true posterior. This is done by means of marginal moment matching steps, which can be computed easily. *Q*($A_{i,}^{T}.$) depends on ***X***, ***U***_·,*i*_, *σ*^2^, and *τ*, as well as 2*N *EP variational parameters. A un over all these costs *O*(*N *min(*N*, *m*)^2^) time, and apart from ***X***, ***U***, the posterior representation is of size *O*(min(*N*, *m*)^2^). A numerically robust implementation for the sparse linear model is challenging and requires some measures not previously proposed for EP.

More specifically, fix a row index *i *and let ***a ***= $A_{i,}^{T}.$. Then, *P*(***a***|*D*) ∝ *N *(***U***_·,*i*_|***Xa***, *σ*^2^***I***) ∏_*j *_*t*_*j *_(*a*_*j*_) with *t*_*j*_(*a*_*j*_) = exp(-*τ*|*a*_*j*_|). The EP approximation has the form *Q*(***a***) ∝ *N*(***U***_·,*i*_|***Xa***, *σ*^2 ^***I***) ∏_*j *_$\tilde{t}$_*j*_(*a*_*j*_) with ${\tilde{t}}_{j}(a_{j})=\exp(b_{j}a_{j}-\frac{1}{2}\pi_{j}a_{j}^{2})$, and comes with 2 *N site parameters ****b***, π. An EP update of the current approximation *Q *focuses on a site *j *∈ {1, ..., *N*}, constructing the distribution $\hat{P}$_*j*_(***a***) ∝ *Q*(***a***)*t*_*j*_(*a*_*j*_)/$\tilde{t}$_*j*_(*a*_*j*_), then adjusting *b*_*j*_, *π*_*j *_such that the new approximation *Q' *matches first and second order moments (mean and covariance) of $\hat{P}$_*j*_. Intuitively, this step is a principled part of the effort of matching these moments of the full posterior. While the latter is not analytically tractable, each EP update actually is. $\hat{P}$_*j *_is non-Gaussian, but since the troublesome factor *t*_*j*_(*a*_*j*_) depends on a single coordinate of ***a ***only, we may still compute mean and covariance efficiently. Note that the EP update can be computed analytically in the Laplace case, but EP can even be used with sites *t*_*i*_(*a*_*i*_) for which this is not possible. In such situations, the non-analytic computation is merely a one-dimensional quadrature, which can be computed by standard numerical techniques. Each EP update works like the inclusion of new evidence *t*_*j*_(*a*_*j*_) in a Bayesian setting, with the difference that in EP, we iterate multiple times over all sites, until convergence. For this reason, we need to divide by $\tilde{t}$_*j*_(*a*_*j*_) in each update, so as to avoid counting site information twice.  Each update affects the posterior approximation *Q*(***a***) globally. Although only *b*_*j*_, *π*_*j *_are modified, this affects all of *Q *due to the presence of the coupling factor *N*(***U***_·,*i*_|***Xa***, *σ*^2 ^***I***). *Q*(***a***) has a representation, whose modification is the dominating computational effort during an EP update. Recall that the sites *t*_*j *_are log-concave. A direct consequence for EP is that each update can actually be done, in that $\hat{P}$_*j *_has a finite covariance, and that the novel *π*_*j *_is non-negative [30]. Empirically, log-concavity seems to imply fast convergence of EP to a close posterior approximation, and a numerically robust behavior can be obtained in a careful implementation.

In the under-determined case *m *<*N *we are principally interested in here, this standard application of EP fails, because the Gaussian coupling factor cannot be normalized as distribution over ***a***. The variant of EP we are using in our experiments here, does come with essentially the same motivation, but some more complicated details. It is described in [29].

Returning to experimental design, the information gain score *S*(u_*_, x_*_|*D*) for an experimental outcome (u_*_, x_*_) is D[*Q' *|| *Q*]. Note that two things happen in *Q *→ *Q'*. Firstly, (u_*_, x_*_) is included, which modifies the Gaussian coupling factor in *Q*. Secondly, the site parameters ***b***, ***π***are updated by EP. For the purpose of scoring, early trials showed that the second step can be skipped in scoring without much loss in performance. Doing so, we see that ***M ***in the equation for D[*Q' *|| *Q*] has the form ***I ***+ x_*_$v_{*}^{T}$, and *S*(u_*_, x_*_|*D*) can be computed very efficiently using a rank one matrix update in our representation of *Q*(***a***). In practice, the effort of scoring even a large number of candidates is clearly dominated by the EP updates after each inclusion.

#### Running Time

The running time for a naive implementation of our method (Laplace prior, experimental design) is *O*(*N*^5^), if *N *experiments are done. Namely, after each experiment, we need to update *N *posterior representations, one for each row of ***A***. For each, we require at least *N *EP updates, one at each Laplace site, and each such update costs *O*(*N*^2^) (at least once *m*, the number of experiments so far, is close to *N*).

This scaling behaviour can be improved by noting that especially during later stages, it will not be necessary to do EP updates for all *N*^2 ^sites after each new experiment. For a row ***a***, we can compute the change in marginal moments of each *Q*(*a*_*i*_) upon including the new observation into the likelihood *P*^(0) ^only. We then do EP updates for *O*(1) sites only, namely the ones with most significantly changed marginals. This cuts the scaling to *O*(*N*^4^).

#### Relations to other Sparse Bayesian Methods

Interestingly, EP for the sparse linear model can be compared directly to the sparse Bayesian learning (SBL) approach [15]. While SBL is formulated in terms of Student-*t *priors, we can do the same scale-mixture decomposition as they do for the Laplace prior [31]. The SBL approach leads to a Gaussian posterior approximation *Q*(***a***) of the same form as in EP, with the difference that in the site approximations $\tilde{t}$_*j*_, the *b*_*j *_parameter is set to zero and eliminated. The presence of the *b*_*j *_parameters in EP is important, because only these guarantee that every possible posterior mean *can *actually be represented in *Q*(***a***).

The *π*_*j *_are chosen in SBL by maximizing the likelihood of the data, integrating out the parameters ***a***. This is a non-convex problem which requires some optimization code, while EP comes with a method of updating *b*_*j*_, *π*_*j *_which can be motivated more directly. The role of log-concavity is also less clear in SBL. A systematic comparison between these approaches is subject to future work. Note that SBL with Student-*t *priors has been applied to gene network recovery [14], although they did not consider experimental design. Furthermore, the Student-*t *distribution is not log-concave, so the true posterior is multimodal, rendering the quality of the Gaussian SBL approximation questionable.

A Markov chain Monte Carlo (MCMC) method for the linear model with Laplace prior is given in [31]. In their approach, the noise variance *σ*^2 ^is inferred together with ***a***, and they give arguments why their sampler should converge quickly, based again on posterior log-concavity. While a direct comparison to our EP variant has not been done, it seems clear that the MCMC approach is much more costly. This may not be a problem for a standard application, but is likely to make the experimental design approach computationally unattractive. In general, while MCMC inference approximations are exact in the limit of large running time, it is very hard even for experts to assess at which point an MCMC estimate can be considered reliable.

#### Incorporating Biological Prior Knowledge

In our method presented so far, we assumed that nothing is known about the network system matrix ***A***, apart from it being sparse. In many applications, substantial additional prior knowledge about ***A ***is available. In this section, we show how some types of such prior knowledge can be incorporated into our method, leading to fewer experiments required for identification. In general, our method can be extended by using additional *sites *beyond the $t_{j}(a_{ij})=\frac{\tau }{2}e^{-\tau \left|a_{ij}\right|}$ coming from the Laplace prior. Such sites must have the form *f*(***w***^*T *^$A_{i,}^{T}.$), where ***w ***∈ ℝ^*N *^and *f*(·) is log-concave.

First, suppose that mRNA degradation rates for some genes are roughly known from independent experiments, say *r*_*i *_for gene *i*. We could either fix *a*_*ii *_= - *r*_*i *_and eliminate this variable, or we could use the factor

$$P(a_{ii})=\frac{\tau }{2}e^{-\tau \left|a_{ii}+r_{i}\right|}$$

with smaller *τ *than usual, which would allow for errors in the knowledge of *r*_*i*_. Using such off-center factors is of course possible in our framework with very minor changes.

Next, suppose that partial connectivity knowledge is available. For example, if there is no influence *j *→ *i*, then *a*_*ij *_= 0, and the corresponding variable can simply be eliminated. If it is known that *j *→ *i *is an activating influence, this means that *a*_*ij *_> *ε *for some *ε *≥ 0. We can incorporate a site ${\text{I}}_{\left\{a_{ij}>\varepsilon \right\}}$ into our method, noting that this is log-concave as an indicator function of a convex set (*ε*, ∞). A better option is to assume that *a*_*ij *_- *ε *has an exponential prior distribution, which also gives rise to a log-concave site.

### Setting Free Parameters

We need to adjust two free parameters: the noise variance *σ*^2^, and the scale *τ *of the Laplace prior. Given some substantial amount of observations, these could be estimated by empirical Bayesian techniques, but this is not possible for experimental design, where we start with very few observations. One may be able to correct initial estimates of *σ*^2^, as more observations are made, and a method for doing so is subject to future work.

There are two sources of noise, *i.e*. non-zero *ε *for observations (***u***, ***x***) and true linearization matrix ***A***. First, the ODE of our simulator is stochastic, and measurement errors are made for ***u***, ***x***. Second, we have systematic deviations between the true non-linear dynamics to ones of the linearization. It is possible to estimate the variance of errors of the first kind without knowing the true ***A ***or performing specific disturbance experiments, by observing fluctuations around the undisturbed steady state. This is not possible for errors of the second kind. However, it is reasonable to assume that a good value for *σ*^2 ^does not change too much between networks with similar biological attributes, so that we can transfer it from a system whose dynamics are known, or for which sufficiently many observations are already available. This transfer was simulated in our experiments by generating 50 networks with data as mentioned above, then estimating *σ*^2 ^from the size of the ***ε***residuals. Note that these additional networks were only used to determine *σ*^2^, for the other experiments we used independent samples from our network generator. The scale parameter *τ *determines the *a priori *expected number of edges in the network. It could be determined similar to *σ*^2^, but a simple heuristic worked just as well in most setups we looked at (the exception was very high noise situations). We need a rough guess of the average node in-degree $\bar{d}$. Then, under the Laplace prior, we expect $\bar{d}$ to be $N{\text{e}}^{-\tau \delta_{e}}$*a priori*. Solving for *τ*, we obtain

$$\tau =-\frac{1}{\delta_{e}}\log\frac{\bar{d}}{N}.$$

We found in practice that our method is quite robust to moderate changes in *τ *and *σ*^2^, as long as the correct order of magnitude is chosen.

### Unobserved variables

Complete gene regulatory networks consist of mRNA concentrations, but also of proteins and metabolites. In typical setups, only (some) mRNA levels are directly measured, and we will discuss here how the unobserved elements of the network influence our network inference. For simplicity, all unobserved quantities will be termed as proteins in this section.

Denote the observed mRNA concentrations by ***x***(*t*) ∈ ℝ^*N *^as before, unobserved protein concentrations by ***y***(*t*) ∈ ℝ^*M*^. Let ***u***(*t*) ∈ ℝ^*N *^be a perturbation vector, which does not affect the unobserved variables. We now have a joint (nonlinear) ODE system for (***x***, ***y***), which is again linearized around its steady state. If time constant perturbations are used, the difference between new and old steady state follows a linear equation (up to noise)

$$\left(\begin{array}{c} u\\ 0 \end{array}\right)=\left(\begin{array}{cc} A & B\\ C & D \end{array}\right)\left(\begin{array}{c} x\\ y \end{array}\right).$$

From this, we deduct ***u ***= (***A ***- ***BD***^-1^***C***)***x***. Thus, given only the ***u ***and ***x ***our algorithm will not recover ***A***, but $\tilde{A}$ = ***A ***- ***BD***^-1^***C***.

We show that $\tilde{A}$ encodes an *effective *gene network in the following sense. If $\tilde{A}$_*ij *_≠ 0, then there exists either a direct link from gene *j *to gene *i *or there is a path from gene *j *to gene *i *which also passes through some proteins in the full gene regulatory network, but not through other observed genes. This is logically equivalent to the statement, that if there is no such path from *j *to *i*, then $\tilde{A}$_*ij *_= 0. However, $\tilde{A}$_*ij *_= 0 does *not *imply that there is no (indirect) connection between *i *and *j*. It could be for example that two protein pathways from *j *to *i *are equally strong, but of opposite influence on gene *i*, and thus cancel each other. To prove that $\tilde{A}$ encodes such an effective network, we first need the following lemma.

**Lemma 1**. *Let ****W ***∈ ℝ^*n*,*n *^*be the weighted adjacency matrix of a directed graph, in that i *← *j has weight w_ij_, and the edge is present iff w_ij _*≠ 0. *Assume that **W **is nonsingular. The following holds: if *(***W***^-1^)*_ij _*≠ 0, *then there exists some directed path j *→ *i*.

*Proof*. We prove the logical converse. For *i *= *j*, there is always a path of length 0 from *i *to *i*, so the lemma makes no statement. For *i *≠ *j*, assume that there is no directed path from *j *to *i*. Let *J *be the set of all nodes reachable by *j *(note that *j *∈ *J*), and let *I *be its complement. *i *∈ *I *by our assumption. Without loss of generality, assume that *J *= {1, ..., |*J*|}, noting that this can always be achieved by renaming nodes, without changing the network. Now,

$$W=\left(\begin{array}{cc} W_{J} & W_{J,I}\\ 0 & W_{I} \end{array}\right).$$

If ***W***_*I*,*J *_was not zero, there would be some element in *I *reachable from *J*, therefore from *j*, so *I *∩ *J *≠ ∅, a contradiction. From the special form of ***W ***we have that |***W***| = |***W***_*J*_||***W***_*I*_|, so that both ***W***_*J*_, ***W***_*I *_are nonsingular. Now,

$$W^{-1}=\left(\begin{array}{cc} W_{J}^{-1} & R\\ 0 & W_{I}^{-1} \end{array}\right),$$

with $R=-W_{J}^{-1}W_{J,I}W_{I}^{-1}$. This proves the lemma.   □

Back to the effective gene network, we have that $\tilde{A}$_*ij *_= ***A***_*ij *_- ∑_*k*,*l *_***B***_*ik*_(***D***^-1^)_*kl*_***C***_*lj*_. Suppose there is no path from *j *→ *i *passing through ≥ 0 proteins only in the full network. Then, ***A***_*ij *_= 0 (no direct gene-gene link). Furthermore, ***B***_*ik*_(***D***^-1^)_*kl*_***C***_*lj *_≠ 0 for some *k, l *would mean a path from gene *j *to protein *l*, then to protein *k *via potentially other proteins (apply the lemma above with ***W ***= ***D***), then to gene *i*. Therefore, all terms in the sum are zero, and $\tilde{A}$_*ij *_= 0.

While we can thus recover an effective network, the knowledge of $\tilde{A}$ does not uniquely determine ***A***, ***B***, ***C***, or ***D***, or in fact even the number *M *of unobserved variables.

## Availability and Requirements

The methods described in the paper are available as a Matlab package at . The code makes use of C++ MEX files for core routines, pre-complied binaries are provided for Windows and Linux 32 bit operating systems. The code is published under the GNU GPL licence, for commercial use please contact the authors.

## Authors' contributions

FS was involved in defining the problem statement, and FS, MS decided on the model solution. FS carried out the numerical experiments and performed the comparison to [3]. MS designed and implemented the computational framework of the approximate inference algorithm. KT proposed to look at the experimental design problem and helped to dicuss the plausibility of the work, as well as relations to other proposed approaches. All authors contributed significantly to the writing of the final manuscript.

## Supplementary Material

Additional file 1 **Implementation details**. The text describes how to sample small-world networks, how the parameters of the simulator were chosen, and describes in detail how the re-implementation of the method [3] was performed. Click here for file
