# Supplementary material for: Experimental design for efficient identification of gene regulatory networks using sparse Bayesian models
Source: BMC Syst Biol. 2007 Nov 16;1:51. doi: 10.1186/1752-0509-1-51 (PMC2233642; doi:10.1186/1752-0509-1-51)
Supplement: Additional file 1 — Implementation details. The text describes how to sample small-world networks, how the parameters of the simulator were chosen, and describes in detail how the re-implementation of the method [3] was performed. [file 1752-0509-1-51-S1.pdf]

Experimental Design for Efficient Identification of Gene Regulatory Networks using Sparse Bayesian Models Supplemental Material

Florian Steinke, Matthias Seeger, Koji Tsuda

1 Sampling small-world networks

Following the description in (Albert and Barabási, 2002) we generate our random *small-world* networks using two steps: first we generate a network with nodes equally distributed on the unit circle and connect each node randomly to 50% of its 4 nearest neighbours. Then we create long range edges by randomly connecting any two nodes. In order to get a directed graph we orient edges with equal probabilities.

For our most commonly used networks of size $N = 50$ nodes showed in-degrees (excluding self-edges) in the range $\{0, ..., 6\}$ (average 2.3).

2 Dynamics of the Simulator

A review of potential dynamics for gene regulatory networks is given in Smolen et al. (2000). Here, the form of the non-linear dynamic model and the parameter ranges were designed in smilarity to the system described in (Kholodenko et al., 2002, Supporting Table 2).

Parameters were drawn randomly, subject to the model producing dynamics with a stable steady state with values in $[0, 10]$. $U[a..b]$ is the uniform distribution between a and b.

Parameter	Description	Range
V_{di}	Max. enzyme rate for degradation	$U[150..500]$
d_i	Max. degradation level	$U[20..70]$
κ_{ij}	Half-saturation / Michaelis constant	$U[20..70]$
n_{ij}	Hill coefficient	$U[1..2]$
V_{si}	Basal rate of expression	$U[3..5]$
A_{ij}	Max. over-expression factor	$U[2..5]$

Typical linearization matrices A obtained at the unperturbed steady state have non-vanishing entries with mean zero and standard deviation 1.1, yet some quite large values do occur.

3 The Method of Tegnér *et.al.*

We first describe the approach of Tegnér et al. (2003) in Bayesian terms, which facilitates a comparison to ours. They start by discretising the space of possible matrices $\boldsymbol{A}$, having a finite number of bins for values of a_{ij}, one of them symmetric around 0. This results in a finite (but large) number of hypotheses for $\boldsymbol{A}$, and they put a uniform prior on allowable matrices: for each gene i, only up to three non-zero a_{ij} are allowed. In other words, the node in-degree is limited to three in their, and also in our comparative experiments here. Their likelihood is an indicator distribution, in that $\boldsymbol{A}$ is *consistent* with the observations iff $\boldsymbol{u} = \boldsymbol{A}\boldsymbol{x} + \boldsymbol{\varepsilon}$ is fulfilled up to a bounded error $\boldsymbol{\varepsilon}$, across all measurements taken. Their posterior is therefore uniform over all (discretized) $\boldsymbol{A}$ consistent with the data and of node in-degree at most three. Experimental design in their method works by next perturbing the gene j for which the variance of a_{ij}'s (outgoing edges) is maximal, under this posterior.

We now give details of our implementation of their method. As (Tegnér et al., 2003) do not explicitly define what a *consistent* solution is, we will state the criterion that we used, in order to make our implementation of their method comparable.

Let us just consider one row of $\boldsymbol{A}$, namely $\boldsymbol{A}_{*,:}$. We assumed that the maximal in-degree is $k = 3$, i.e. there are at most 3 non-zero entries in $\boldsymbol{A}_{*,:}$ apart from the diagonal entry a_{**}. The non-zero entries are quantized into bins of equal width $\Delta_{\boldsymbol{A}}$ and with means $\bar{a}_j$ (j being the index of the bin). Symmetric around zero an interval of width $2\Delta_{\boldsymbol{A}}$ is excluded, for these entries are assumed to be zero and do not represent edges. $\boldsymbol{A}_{*,:}$ is then fully described by up to three tuples of one bin index j and one column index i each, *i.e.* by $D_* = \{(j(k), i(k))\}_{k \leq 3}$. We will assume that the measurement error of any component of $\boldsymbol{x}$ is at most Δ_x, that the maximal absolute value of $\boldsymbol{x}$ is x_{max}, and that the diagonal entry a_{**} is known exactly. We consider the row $\boldsymbol{A}_{*,:}$ given through a descriptor D_* as consistent with a measurement $(u_*, \boldsymbol{x}) \in \mathbb{R} \times \mathbb{R}^N$ if the value u_* falls into the following range

$$a_{**}\left(x_* \pm \Delta_x\right) \pm \Delta_x + \sum_{k=1}^{|D_*|} \left(\bar{a}_{j(k)} \left(x_{i(k)} \pm \Delta_x \right) \pm \frac{\Delta_{\boldsymbol{A}}}{2} x_{i(k)} \right) \pm (3 - |D_*|) \Delta_{\boldsymbol{A}} x_{max}.$$

This considers quantisation errors in the matrix entries of $\boldsymbol{A}$ and measurement errors in $\boldsymbol{x}$ and $\boldsymbol{u}$. The last term helped to improve results, and accounts for entries in $\boldsymbol{A}$ that are smaller than $\Delta_{\boldsymbol{A}}$ but may still represent an edge.

Given this criterion our implementation was quite simple: after the first random experiments, all possible row descriptors are checked whether they are consistent, and if so, were stored in an array. After each inclusion, only this array is parsed to detect row descriptors which have become inconsistent through the last experiment.

References

Albert, R. and Barabási, A.-L. (2002). Statistical mechanics of complex networks. *Rev. Mod. Phys.*, 74(1):47–97.

Kholodenko, B. N., Kiyatkin, A., Bruggeman, F. J., Sontag, E., Westerhoff, H. V., and Hoek, J. B. (2002). Untangling the wires: A strategy to trace functional interactions in signaling and gene networks. *PNAS*, 99(20):12841–12846.

Smolen, P., Baxter, D., and Byrne, J. (2000). Mathematical Modeling of Gene Networks. *Neuron*, 26:567–580.

Tegnér, J., Yeung, M. K. S., Hasty, J., and Collins, J. J. (2003). Reverse engineering gene networks: Integrating genetic perturbations with dynamical modeling. *PNAS*, 100(10):5944–5949.
